# Concurrent versus Sequential Sorafenib Therapy in Combination with Radiation for Hepatocellular Carcinoma

**DOI:** 10.1371/journal.pone.0065726

**Published:** 2013-06-06

**Authors:** Aaron T. Wild, Nishant Gandhi, Sivarajan T. Chettiar, Khaled Aziz, Rajendra P. Gajula, Russell D. Williams, Rachit Kumar, Kekoa Taparra, Jing Zeng, Jessica A. Cades, Esteban Velarde, Siddharth Menon, Jean F. Geschwind, David Cosgrove, Timothy M. Pawlik, Anirban Maitra, John Wong, Russell K. Hales, Michael S. Torbenson, Joseph M. Herman, Phuoc T. Tran

**Affiliations:** 1 Department of Radiation Oncology & Molecular Radiation Sciences, Sidney Kimmel Comprehensive Cancer Center, Johns Hopkins University School of Medicine, Baltimore, Maryland, United States of America; 2 Department of Pharmacology and Molecular Sciences, Johns Hopkins University School of Medicine, Baltimore, Maryland, United States of America; 3 Department of Radiology, Johns Hopkins University School of Medicine, Baltimore, Maryland, United States of America; 4 Department of Oncology, Sidney Kimmel Comprehensive Cancer Center, Johns Hopkins University School of Medicine, Baltimore, Maryland, United States of America; 5 Department of Surgery, Johns Hopkins University School of Medicine, Baltimore, Maryland, United States of America; 6 Department of Pathology, Johns Hopkins University School of Medicine, Baltimore, Maryland, United States of America; 7 Department of Urology, Johns Hopkins University School of Medicine, Baltimore, Maryland, United States of America; National Taiwan University, Taiwan

## Abstract

Sorafenib (SOR) is the only systemic agent known to improve survival for hepatocellular carcinoma (HCC). However, SOR prolongs survival by less than 3 months and does not alter symptomatic progression. To improve outcomes, several phase I-II trials are currently examining SOR with radiation (RT) for HCC utilizing heterogeneous concurrent and sequential treatment regimens. Our study provides preclinical data characterizing the effects of concurrent versus sequential RT-SOR on HCC cells both *in vitro* and *in vivo*. Concurrent and sequential RT-SOR regimens were tested for efficacy among 4 HCC cell lines *in vitro* by assessment of clonogenic survival, apoptosis, cell cycle distribution, and γ-H2AX foci formation. Results were confirmed *in vivo* by evaluating tumor growth delay and performing immunofluorescence staining in a hind-flank xenograft model. *In vitro*, concurrent RT-SOR produced radioprotection in 3 of 4 cell lines, whereas sequential RT-SOR produced decreased colony formation among all 4. Sequential RT-SOR increased apoptosis compared to RT alone, while concurrent RT-SOR did not. Sorafenib induced reassortment into less radiosensitive phases of the cell cycle through G_1_-S delay and cell cycle slowing. More double-strand breaks (DSBs) persisted 24 h post-irradiation for RT alone versus concurrent RT-SOR. *In vivo*, sequential RT-SOR produced the greatest tumor growth delay, while concurrent RT-SOR was similar to RT alone. More persistent DSBs were observed in xenografts treated with sequential RT-SOR or RT alone versus concurrent RT-SOR. Sequential RT-SOR additionally produced a greater reduction in xenograft tumor vascularity and mitotic index than either concurrent RT-SOR or RT alone. In conclusion, sequential RT-SOR demonstrates greater efficacy against HCC than concurrent RT-SOR both *in vitro* and *in vivo*. These results may have implications for clinical decision-making and prospective trial design.

## Introduction

Hepatocellular carcinoma (HCC) is a major global cause of mortality, accounting for the 3^rd^ most cancer-related deaths worldwide [1]. The incidence of HCC in the United States has tripled over the past two decades [2] and is expected to continue rising in coming years as chronic hepatitis C infections contracted during the 1960s and 70s reach an incubation period of 40–50 years [3]–[5]. Meanwhile, the 5-year survival rate has remained below 12% [6]. The dismal prognosis of HCC derives from diagnosis at an advanced, inoperable stage in more than 70% of cases and, until recently, an absence of systemic agents demonstrating meaningful activity against the disease [7].

Two phase 3 studies, however, have now shown sorafenib to be the first systemic agent capable of improving survival for HCC [8], [9]. Sorafenib is an oral multikinase inhibitor that has been observed to act against a variety of cancer cell lines through slowing of cellular proliferation, increased apoptosis, and inhibition of angiogenesis [10]–[15]. These effects are mediated by targeted inhibition of several kinases, including B-Raf, c-Raf, VEGFR2, VEGFR3, platelet-derived growth factor receptor-β, fibroblast growth factor receptor 1, Flt3, c-KIT, RET, and p38α [10], [16]. The frequency of RAS/RAF/MAPK pathway overexpression in HCC (>90% of specimens) [17] and the typically high vascularity of hepatomas [18], [19] provide a sound rationale for the activity displayed by sorafenib against HCC.

While sorafenib has rapidly become accepted as first-line treatment for locally advanced and metastatic HCC, the survival benefit remains modest at less than 3 months and sorafenib imparts no delay in time to symptomatic progression compared to placebo [8], [9], [20]. Strategies to improve clinical efficacy have focused on combining sorafenib with systemic chemotherapy [21]–[24], trans-arterial chemoembolization [25]–[27], and, most recently, radiation. Although no prospective studies have yet been reported, there are currently several ongoing phase I-II trials that employ sorafenib and radiation concurrently, sequentially, or both (Table 1). Meanwhile, no preclinical study to date has examined the effects of combined sorafenib-radiation treatment on HCC cell lines in both the *in vitro* and *in vivo* settings, possibly in part accounting for the heterogeneity of experimental clinical regimens. Our goal, therefore, was to study sorafenib combined with radiation in the preclinical setting so as to provide evidence for an optimal sequencing strategy in upcoming large-scale trials for HCC.

**Table 1:** Active clinical trials currently registered on ClinicalTrials.gov that involve the administration of sorafenib and radiation for hepatocellular carcinoma (HCC).

Study Name	Phase	Identifier	Location	Start DateCompletion Date	Targeted Enrollment	Treatment Schedule
Study of combined sorafenib with radiotherapy in patients with advanced HCC	2	NCT01328223	China Medical University Hospital	September 2010December 2012	45	Concurrent+Sequential
Radiation therapy with sorafenib for TACE-resistant HCC	1	NCT01618253	Medical College of Wisconsin	June 2012June 2016	30	Concurrent
Stereotactic radiation therapy and sorafenib in the treatment of HCC	0	NCT01005875	University of Alabama at Birmingham	November 2009November 2012	10	Sequential
Proton beam radiotherapy plus sorafenib versus sorafenib for patients with HCC exceeding San Francisco criteria	2/3	NCT01141478	Loma Linda University	August 2010June 2015	220	Concurrent+Sequential
Sorafenib-RT in treating HCC (SHEP)	2	NCT00892658	University Health Network, Toronto	January 2009January 2013	44	Concurrent+Sequential
Safety study of sorafenib following combined therapy of radiation and TACE for liver cancer	1/2	NCT00999843	Fudan University	October 2009October 2012	30	Sequential
Study of SIR-Spheres plus sorafenib as 1st line treatment for non-resectable primary HCC	1/2	NCT00712790	Singapore Clinical Research Institute	June 2008June 2010	35	Not specified

If available, the treatment schedule is specified in the right-most column; all sequential schedules consisted of radiotherapy first followed by sorafenib.

## Experimental Procedures

### Ethics Statement

This study was carried out in strict accordance with the recommendations in the Guide for the Care and Use of Laboratory Animals of the National Institutes of Health. The protocol was approved by the Animal Care and Use Committee of the Johns Hopkins University (Protocol Number: MO09M331). All efforts were made to minimize suffering.

### Cell Lines and Cell Culture

Three representative, well-characterized human HCC cell lines obtained from ATCC and one mouse MYC-induced HCC cell line were used: HepG2 (wild-type p53), HuH7 (Y220C-mutated p53; p21 deficient), Hep3b (p53 and pRb deficient; hepatitis B virus positive), and HCC-4-4 (murine; MYC-induced) [28]. Cells were grown and maintained in DMEM medium supplemented with 10% FBS and 1% penicillin-streptomycin. All cells were incubated at 37°C in humidified 5% CO_2_. Cells were sub-cultured at 70–80% confluence and all experiments were carried out with the cells in an exponential growth phase.

### Sorafenib treatment

Sorafenib (tosylate) was purchased from Selleck Chemicals (Houston, TX). The drug was stored at −20°C in DMSO at a 5 mM stock solution for *in vitro* studies. For *in vivo* experiments, the drug was formulated in 1∶1 cremophor/ethanol, stored at 24 mg/mL stock concentration at room temperature, diluted with sterile water to a concentration of 6 mg/mL for treatment, and injected at a dose of 60 mg/kg, as has been used previously [29].

### Radiation therapy

For *in vitro* experiments, cells were irradiated with 0–6 Gy using GammaCell with ^137^Cs source at 50 cGy/min. For *in vivo* experiments, mice were treated using the Small Animal Radiation Research Platform (SARRP) [30]. The tumors were irradiated with a circular beam of 1-cm diameter with 3 consecutive daily fractions of 3 Gy.

### Clonogenic assay

Cells in exponential growth phase were counted and plated in 10-cm dishes containing 10 mL medium each. Depending on the cell type, drug concentration and radiation dose, 150–10,000 cells were plated. For concurrent treatment, cells were allowed to attach and sorafenib 5 µM was added to the medium 24 h after plating. Twenty-four hours following the addition of sorafenib, radiation was delivered. Sorafenib-containing medium was removed and replaced with normal growth medium 24 h after irradiation. For sequential treatment, cells were allowed to attach and radiation was delivered 24 h after plating. Twenty-four hours following irradiation, sorafenib 5 µM was added to the medium. Sorafenib was removed 48 h later and replaced with normal growth medium. Medium was changed every 4–5 days for both concurrent and sequential experiments. Colonies were stained and counted 10–14 days after irradiation by fixing and staining with a solution of 0.1% Gentian violet in 1∶1 methanol/deionized water. Colonies were counted under an inverted phase contrast microscope (Nikon Instruments Inc., Melville, NY) with a colony defined as comprising at least 50 cells. Surviving fraction was calculated as a function of plating efficiency. All arms were done in triplicate and repeated at least three times to ensure reproducibility.

### Cell cycle analysis

For experiments with unsynchronized cells, 100,000–300,000 cells were seeded per well in 6-well plates. Sorafenib was added 24 h after plating. For experiments with synchronized cells, cells were allowed to attach in normal growth media for 24 h, serum starved for 48 h (0% serum), then grown in the presence of 10% serum and aphidicolin (2 µg/mL) for 24 h before being released into normal growth medium (10% serum, without aphidicolin) containing sorafenib. At various time points after adding sorafenib, cells were detached, washed with phosphate-buffered saline (PBS) and fixed with chilled 70% ethanol. Cells were pelleted and washed in PBS+1% BSA, then treated with 20 µg/mL RNAse-A with 10 µg/mL propidium iodide for 2 h. DNA content was analyzed with a FACSCalibur (BD Biosciences, Franklin Lakes, NJ) and FlowJo analysis software (Tree Star, Ashland, OR).

### Apoptosis assay

Apoptosis assays were performed using the FITC Annexin V/Dead Cell Apoptosis Kit with Annexin V-FITC and Propidium Iodide (Invitrogen, Carlsbad, CA) for flow cytometry. Cells were seeded at 100,000–300,000 per well in a 6-well plate and treated according to one of the 5 treatment arms (vehicle control—incubation with DMSO for 48 hours; sorafenib alone—incubation with 5-µM sorafenib for 48 hours; radiation alone—incubation with DMSO for 48 hours with irradiation at 24-hour midpoint; concurrent—incubation with 5-µM sorafenib for 24 hours with irradiation at 24-hour midpoint; sequential—incubation with DMSO for 24 hours, irradiation, followed by incubation with 5-µM sorafenib for 24 hours). All irradiation doses were single fractions of 6 Gy. After treatment, cells were detached using trypsin-EDTA 0.25%, washed in PBS, suspended in binding buffer, and FITC Annexin V (5 µL stock/100 µL buffer) and propidium iodide (100 ng/100 µL buffer) were added. After incubating for 15 m at room temperature in the dark, cells were analyzed with a FACSCalibur (BD Biosciences) and FlowJo software (Tree Star). Unstained and single-stained cells were used to choose the correct gating. Experiments were done at least twice in triplicate to ensure reproducibility.

### Immunoblot analysis

Cells were grown to sub-confluence in 10-cm dishes, then sorafenib (1–10 µM) was added and cells were harvested and homogenized 24 h later (when probing for phospho-ERK1/2 and ERK1/2). Alternatively, when probing for phospho-p53 (Cell Signaling Technology, Danvers, MA) and p21 (Calbiochem, Billerica, MA), cells were treated according to one of the 5 treatment arms (vehicle control—incubation with DMSO for 12 hours; sorafenib alone—incubation with 5-µM sorafenib for 12 hours; radiation alone—incubation with DMSO for 12 hours with irradiation at 6-hour midpoint; concurrent—incubation with 5-µM sorafenib for 12 hours with irradiation at 6-hour midpoint; sequential—incubation with DMSO for 6 hours, irradiation, followed by incubation with 5-µM sorafenib for 6 hours) and then were harvested and homogenized. 50–100 µg of total protein was loaded into wells of an 8–12% TRIS-HCL Ready Gel (BioRad, Hercules, CA). Protein was separated and transferred onto polyvinylidene fluoride (BioRad), then blocked for 1 h using 5% BSA in TBST (Tris-buffered Saline supplemented with 0.1% Tween-20). Phospho-antibodies were incubated with 5% BSA in TBST, while other antibodies were incubated with 5% milk in TBST. Membranes were probed with antibodies for phospho-p44/42 MAPK (phospho-Erk1/2), p44/42 MAPK (Erk1/2), phospho-S6, and actin (all Cell Signaling Technology) and subsequently with horseradish peroxidase-labeled mouse anti-rabbit secondary antibodies (Sigma-Aldrich, St. Louis, MO). Each antibody incubation step was followed by 3–4 TBST washes. The secondary antibody was then coupled with GE ECL Plus kit (GE Healthcare Life Sciences, Uppsala, Sweden) and protein levels detected using autoradiography films (Denville Scientific, South Plainfield, NJ). Experiments were done at least twice to ensure reproducibility.

### Immunofluorescence

Cells were plated on poly-L-lysine-coated (13.3 mg/mL) cover glass and incubated for 24 h at 37°C in 5% CO_2_. 5-µM sorafenib was added and radiation delivered 24 h later. Cells were fixed at 30 m and 24 h post-irradiation with 4% paraformaldehyde in PBS. After washing with PBS, cells were permeabilized for 15 m with PBST (PBS with 0.1% Triton X-100). The cells were then blocked with 2% FBS/3% BSA in PBS for 30 m and incubated at room temperature for 1 h with primary antibody (1∶250) diluted in PBS. After washing with PBS, the cells were incubated with an AlexaFluor 488-conjugated secondary antibody (1∶300; Molecular Probes, Eugene, OR) for 1 h at room temperature. Cells were washed in PBS and coverslips stained with DAPI prior to mounting. Fluorescent images were captured using a confocal microscope (Carl Zeiss AG, Oberkochen, Germany). The cells were probed with primary antibodies for γ-H2AX (Millipore, Billerica, MA).

### Mouse xenograft model and tumor growth delay experiments

Female athymic nude mice (Harlan, Indianapolis, IN) were maintained under pathogen-free conditions and given food/water *ad libitum* in accordance with Johns Hopkins Animal Care and Use Committee guidelines. Mice were injected subcutaneously in both flanks with 3×10^6^ HepG2 cells in 100 µL of Hank's balanced salt solution and Matrigel (Invitrogen) mixed 1∶1. Once tumors reached 100 mm^3^, 5–6 mice were randomly assigned to each of the five treatment arms as follows: (1) no treatment (sham injections on days 1–5); (2) sorafenib alone (injection of sorafenib on days 1–5); (3) radiation alone (3 Gy×3 fractions on days 1–3); (4) concurrent treatment (injection of sorafenib on days 1–5 with 3 Gy×3 delivered on days 2–4); and (5) sequential treatment (3 Gy×3 fractions on days 1–3 followed by injection of sorafenib on days 4–8). Sorafenib was dosed at 60 mg/kg intraperitoneally in all cases. Tumors were measured three times weekly until reaching quadruple their pre-treatment volume. Tumor volume was calculated using the formula for volume of an ellipsoid: *length×width×height×π/6*.

### Immunofluorescence

Mice with established flank tumors from each of treatment arms (1) through (5) above were sacrificed after days 1–3 of treatment as outlined above; for arms (3), (4), and (5) in which radiation was a component of therapy, mice were sacrificed 1 h after radiation delivery. For arm (5), an abbreviated sequential course of sorafenib therapy was administered as one injection given immediately following the second consecutive daily 3-Gy fraction of radiation prior to tumor harvesting 1 hour later. This harvesting schedule was used for γ-H2AX immunofluorescence staining to maintain consistency with harvesting timing for the radiation alone and concurrent treatment arms. Therefore, due to the transient nature of double-stranded breaks and the close relationship they bear to timing of irradiation, rather than delay harvesting by 3 days following irradiation to allow 3 daily doses of sorafenib in the sequential arm, for γ-H2AX immunofluorescence only (*i.e.*, not for Ki-67 and CD31) these animals received an abbreviated sequential treatment course consisting of immediate injection of sorafenib following the second fraction of radiation and tumor harvesting 1 hour later. This limitation in assay design should be considered when interpreting the γ-H2AX immunofluorescence data presented below. Tumors were harvested, fixed in 10% formalin for 3 days, then transferred to PBS, fixed in paraffin, and sectioned by the Johns Hopkins Tissue Core Facility. Sections were stained for CD31 (Abcam, Cambridge, MA), γ-H2AX (Cell Signaling), and Ki67 (Abcam). The number of positively staining foci were counted and compared for at least five randomly chosen high power fields per tumor.

### Statistics

Error bars included in graphical figures represent standard error of the mean. Two-tailed unpaired Student's t-test was used to compare apoptosis assay, cell cycle analysis, and CD31 immunohistochemistry results between treatment arms. Fisher's exact test was used to compare γ-H2AX foci analysis and Ki-67 immunohistochemistry results between treatment arms. Clonogenic survival curves were fitted with a linear quadratic model using SPSS Statistics version 19 using a least squares fit, weighted to minimize the relative distances squared, and compared using the extra-sum of squares *F* test. Mean inactivation doses were determined using the method of Fertil [31] and enhancement ratios calculated as the ratio of the mean inactivation dose for control versus sorafenib-treated arms as described by Morgan [32]. A value significantly >1 indicates radiosensitization. Tumor growth delay assay results were compared by two distinct methods: (1) by using the log-rank test to compare median quadrupling times after creating a Kaplan-Meier plot using quadrupling in volume as the event of interest; and (2) by comparing quadrupling times between all tumors in two arms using the Mann-Whitney U test. A two-sided alpha value of ≤0.05 was considered significant in all cases.

## Results

### Concurrent treatment with sorafenib and radiation resulted in radioprotection of HCC cell lines *in vitro*

**Figure 1:**
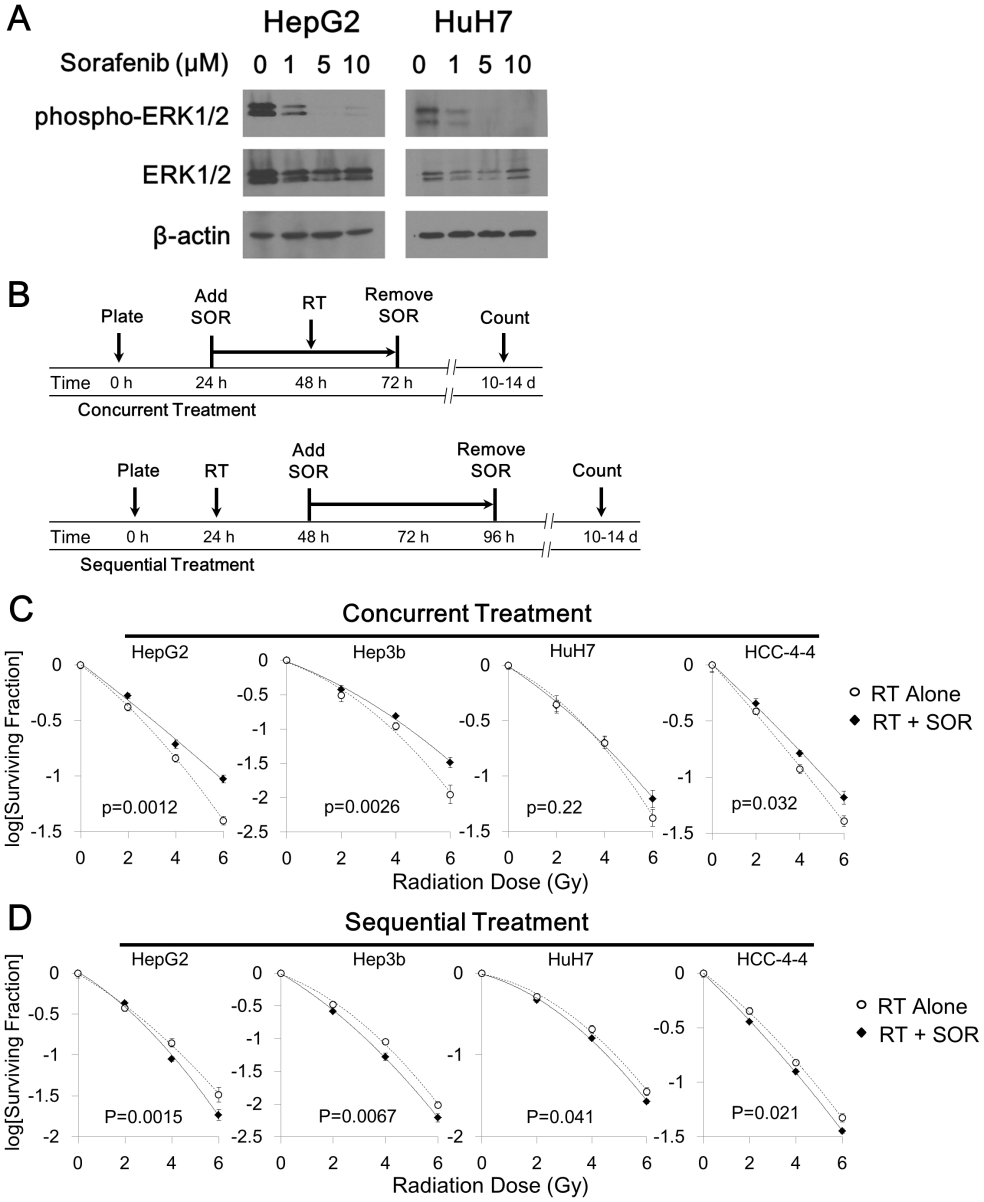
Schedule-dependent effects of combining radiation (RT) and sorafenib (SOR) against HCC cell lines *in vitro*. (A) Immunoblotting for phospho-ERK showed downregulation after 24 hours of SOR treatment in the HepG2 and HuH7 cell lines in a dose-dependent manner. (B) Schematic diagrams depicting the specific timing of concurrent versus sequential treatment with combined RT-SOR are shown. (C) Concurrent treatment with 5 µM SOR plus RT resulted in radioprotection in 3 of 4 HCC cell lines and had no effect on radiosensitivity in the fourth. (D) Sequential treatment with RT plus 5 µM SOR resulted in decreased colony formation in all 4 HCC cell lines. All best-fit curves are second-degree polynomials derived from the linear-quadratic model and have an R^2^ value greater than 0.99.

As shown previously [10], [11], treatment of HCC cells with sorafenib for a period of 24 hours inhibited intracellular signal transduction through the MAP kinase pathway, as demonstrated by Western blotting for phospho-ERK (Fig. 1A). Clonogenic survival assays were performed to determine the direct effect of concurrent sorafenib therapy on radiation sensitivity of HCC cell lines *in vitro* (Fig. 1B,C). We chose to examine the effects of 5 µM SOR throughout, as this concentration is at the lower end of the clinically relevant range achievable in plasma (5–15 µM) [33]. Contrary to our initial hypothesis that posited sorafenib as a potential radiosensitizer, treatment with sorafenib resulted in improved ability to form colonies after irradiation in three of four cell lines, indicating radioprotection. Enhancement ratios (ER) were calculated to be 0.85, 0.90, and 0.92 for HepG2, Hep3b, and HCC-4-4 cell lines, respectively (all p<0.05). The fourth cell line (HuH7) did not demonstrate any difference in radiation sensitivity with concurrent sorafenib treatment (ER = 0.96; p = 0.22).

### Sequential treatment with radiation followed by sorafenib did not result in radioprotection of HCC cells *in vitro*

To investigate whether the results of concurrent sorafenib-radiation treatment were schedule-dependent, the colony forming capacity of each of the four cell lines was then assessed using a sequential radiation and sorafenib regimen (Fig. 1B,D). This treatment schedule consistently resulted in mildly decreased colony formation, with ER of 1.05, 1.07, 1.06, and 1.04 for HepG2, Hep3b, HCC-4-4, and HuH7, respectively (all p<0.05). With the possible exception of HepG2, the shape of the clonogenic survival curves was not noticeably changed by sequential treatment with sorafenib compared with vehicle control, implying that the sequential administration of sorafenib does not alter the radiosensitivity of HCC cell lines nor result in any synergism with radiation therapy. Rather, these observations suggest that sequential radiation-sorafenib treatment has a slight additive inhibitory effect on colony formation *in vitro* and avoids the potential radioprotective effect observed with a concurrent sorafenib-radiation schedule.

### Sequential radiation followed by sorafenib increases apoptosis compared to radiation alone *in vitro*, whereas concurrent treatment shows apoptotic rates similar to those observed with radiation alone

**Figure 2:**
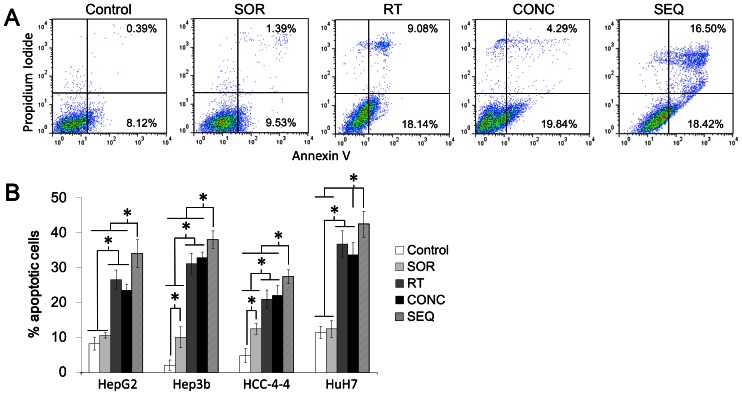
Sorafenib given sequentially with radiation increases the proportion of cells undergoing apoptosis compared to RT alone *in vitro*, while concurrent sorafenib-radiation does not. (A) Cells were treated according to one of the 5 treatment arms (control—incubation with DMSO for 48 hours; sorafenib alone (SOR)—incubation with 5-µM sorafenib for 48 hours; radiation alone (RT)—incubation with DMSO for 48 hours with irradiation at 24-hour midpoint; concurrent (CONC)—incubation with 5-µM sorafenib for 24 hours with irradiation at 24-hour midpoint; sequential (SEQ)—incubation with DMSO for 24 hours, irradiation, followed by incubation with 5-µM sorafenib for 24 hours). All irradiation doses were single fractions of 6 Gy. After treatment, cells were assessed for Annexin V-FITC and propidium iodide using flow cytometry. Representative data for HepG2 cells is shown for the four treatment arms: *from left to right*—Control, SOR, RT, CONC, and SEQ. Sample data for the remaining 3 cell lines can be found in Figure S1A–C. (B) Cells in the early phases of apoptosis (annexin V high, propidium iodide low; quadrant II) and cells in the late phases of apoptosis (annexin V high and propidium iodide high) are summed together and plotted as “% apoptotic cells” with SEM for all 4 cell lines. Asterisks represent statistically significant differences between the treatment groups by Student's *t-*test. All experiments were done in triplicate and repeated.

In order to explore possible mechanisms that could account for the sorafenib-mediated radioprotection observed in colony formation assays, we examined the degree of apoptosis in HCC cell lines after administration of sequential (delivery of a 6-Gy radiation dose followed by 24 h treatment with sorafenib) and concurrent (delivery of a 6-Gy radiation dose at the halfway point of 48-h incubation with sorafenib) treatment regimens, as well as for the appropriate control regimens (radiation alone, sorafenib alone, and vehicle control). Representative plots for HepG2 cells are displayed in Fig. 2A and for the three other cell lines in Figure S1, while a summary of the apoptosis assay results are depicted graphically in Figure 2B. The percentages of cells demonstrating an Annexin V (AV) high/propidium iodide (PI) low staining pattern indicative of early apoptosis (quadrant II) and an AV high/PI high staining pattern indicative of late apoptosis (quadrant III) were summed to yield the total number of apoptotic cells (Fig. 2B).

In all four cell lines, the percentage of cells undergoing apoptosis was greatest following sequential treatment, with absolute increases in apoptotic cells of 5–7% compared to concurrent treatment and radiation alone (all p<0.05, Student's t-test; Figure 2A, 2B, and Figure S1A–C). There were no significant differences in the percentage of cells undergoing apoptosis after treatment with concurrent sorafenib-radiation versus radiation alone (all p>0.05, Student's t-test). Although two cell lines showed a significant increase in apoptosis after treatment with sorafenib alone (Hep3b and HCC-4-4; p = 0.01 and 0.01 compared to vehicle control, respectively, Student's t-test), a significant difference was not observed between concurrent sorafenib-radiation and radiation alone (p = 0.31 and 0.29, respectively, Student's t-test). These experiments were repeated at additional time points of 48 and 72 h following the delivery of radiation, but again no difference was seen between radiation alone and concurrent sorafenib-radiation (data not shown). Collectively, these data suggest that sorafenib treatment alone may increase *in vitro* apoptosis in a cell line specific fashion, but does not augment apoptosis when given concurrently with radiation compared to radiation alone.

Interestingly, among all four cell lines, treatment with concurrent sorafenib-radiation compared to radiation alone reduced the proportion of cells in late apoptosis and increased the proportion of cells in early apoptosis, though, as stated above, the total proportion of cells undergoing either early or late apoptosis remained similar. These data suggest that sorafenib may slow the progression of irradiated HCC cells through apoptosis, possibly allowing more cells adequate time to recover and avert apoptosis in congruence with our colony formation assay results.

### Sorafenib caused G_1_-S delay and cell cycle slowing in HCC cell lines

**Figure 3:**
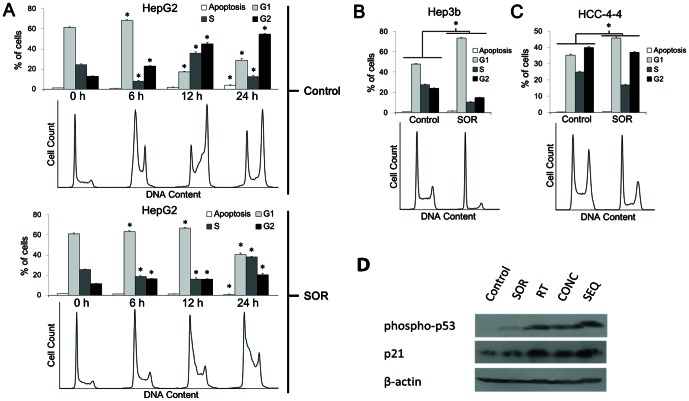
Mechanism of sorafenib-mediated radioprotection *in vitro*. HepG2 cells were synchronized then re-fed with complete medium (10% serum) either containing 5 µM sorafenib (SOR) or vehicle control (DMSO). (A) Percent of cells in G_1_, S, and G_2_ phases with SEM is plotted for control and SOR arms, with corresponding histograms generated from flow cytometry data analysis shown below. Treatment with SOR caused a G_1_-S delay and cell cycle slowing in synchronized HepG2 cells, causing more cells to be in G_1_-S versus G_2_-M when radiation would be delivered 24 h after beginning incubation with SOR. (B & C) Unsynchronized Hep3b and HCC-4-4 cells were exposed to SOR or vehicle control for 24 h and then fixed with ethanol for cell cycle analysis. Percent of cells in G_1_, S, and G_2_ phases with SEM is plotted for control and SOR arms, with corresponding histograms generated from flow cytometry data analysis shown below. Treatment with SOR caused a G_1_-S delay in both cell lines and reduced the number of cells in G_2_-M when radiation would be delivered at 24 h after beginning incubation with SOR. Asterisks denote significant differences between corresponding columns in the control and SOR arms for each cell line by Student's *t-*test. Data for the HuH7 cell line is not shown because it was found to exhibit polyploidy; these data are displayed in Figure S2. Data for unsynchronized HepG2 cells are also shown in Figure S2. All experiments were done in triplicate and repeated. (D) Immunoblotting for phospho-p53 and p21 after treatment of HepG2 cells with each of the 5 different treatment arms (control—incubation with DMSO for 12 hours; SOR—incubation with 5-µM sorafenib for 12 hours; RT—incubation with DMSO for 12 hours with irradiation at 6-hour midpoint; CONC—incubation with 5-µM sorafenib for 12 hours with irradiation at 6-hour midpoint; SEQ—incubation with DMSO for 6 hours, irradiation, followed by incubation with 5-µM sorafenib for 6 hours). All irradiation doses were single fractions of 6 Gy. Corresponding immunoblot data for the remaining 3 cell lines can be found in Figure S2C.

Sorafenib treatment effects on the cell cycle of the four HCC lines were examined (Fig. 3). HuH7 exhibited polyploidy and therefore was not analyzed further (Fig. S2). Unsynchronized Hep3b and HCC-4-4 cells following incubation with sorafenib for 24 h demonstrated a G_1_-S delay in response to sorafenib (Fig. 3B,C). For Hep3b, 73% (SD 2%) of sorafenib-treated cells were in G_1_ phase after 24 h as compared to only 48% (SD 1%) of untreated cells (p<0.00001 by Student's t-test) (Fig. 3B). Likewise, for HCC-4-4 at 24 h, 46% (SD 1%) of cells treated with sorafenib were in G_1_ versus only 35% (SD 1%) of untreated cells (p<0.00001, Student's t-test) (Fig. 3C). Unsynchronized HepG2 cells demonstrated a less pronounced effect on the cell cycle perhaps consistent with generalized cell cycle slowing (Fig. S2). To assess the effects of sorafenib on HepG2 cells further, we synchronized HepG2 cells and then analyzed after incubation with sorafenib for 0, 6, 12, and 24 h (Fig. 3A). Sorafenib treatment caused an overt G_1_-S delay, with the majority of cells (67%, SD 2%) remaining in G_1_ phase at 12 h after treatment with sorafenib versus only 17% (SD 1%) of untreated cells (p<0.00001, Student's t-test). As suspected with unsynchronized HepG2 cells, sorafenib treatment resulted in generalized cell cycle slowing that could easily be observed following synchronization with 79% (SD 4%) of cells treated with sorafenib remaining in G_1_ or S phase at 24 h versus 41% (SD 2%) of untreated cells (p<0.00001, Student's t-test). Altogether, sorafenib treatment resulted in cell cycle reassortment and cell cycle delay of HCC cells.

One possible mechanism behind the sorafenib-mediated G_1_-S delay observed is cell cycle arrest through activation of p53 and expression of downstream effector proteins, such as p21. In order to investigate this possibility, we performed immunoblotting for phospho-p53 and p21 in cells that had been treated with sorafenib compared to vehicle control (Figure 3D, Figure S2C). In all 4 cell lines there was at least a slight increase in phospho-p53 expression following treatment with sorafenib compared to vehicle control. Similarly, three of the four cell lines (HepG2, HuH7, and HCC-4-4) appeared to display higher p21 expression after treatment with sorafenib versus vehicle control, while the fourth cell line (Hep3b) showed inconclusive results demonstrating approximately equal p21 expression levels despite markedly increased phospho-p53 expression in cells treated with sorafenib. These data suggest that activation of p53 and increased expression of its downstream effector proteins may play a role in the G_1_-S delay mediated by sorafenib, although more investigation is required.

### Concurrent treatment with sorafenib and radiation resulted in reduced persistence of double-strand breaks than radiation alone *in vitro*

**Figure 4:**
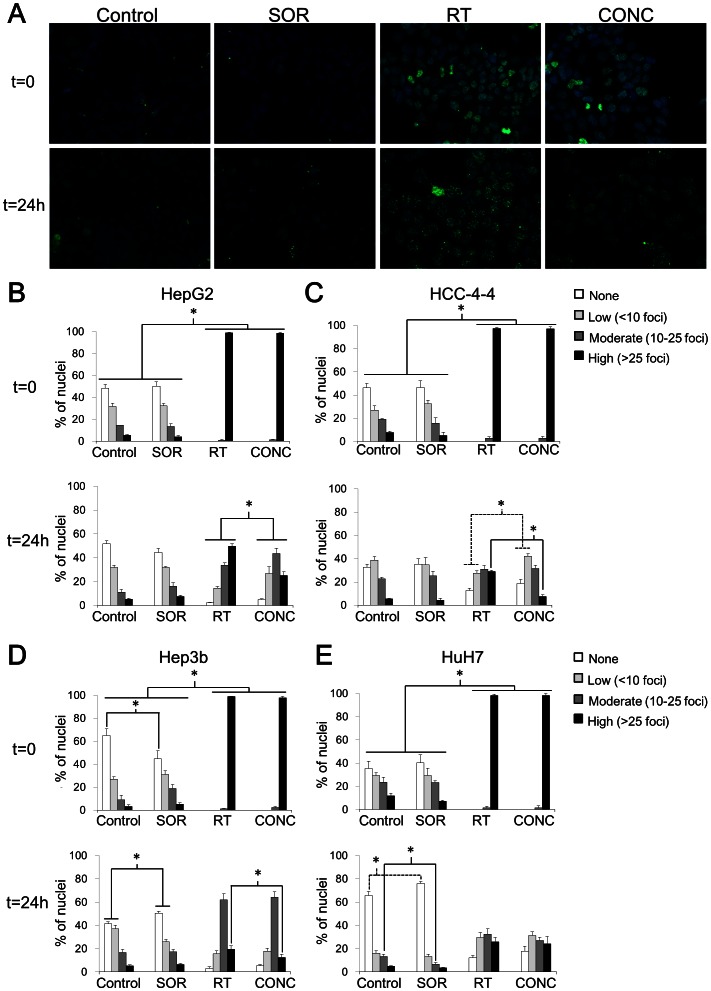
Mechanism of sorafenib-mediated radioprotection. Immunostaining for γ-H2AX foci, and then staining for DAPI were performed as detailed in Materials and Methods (note: the t = 0 time point actually represents cells that were fixed at 30 minutes post-irradiation). Fluorescent images were captured at 40× using a confocal microscope with uniform exposures of 50 ms for DAPI and 900 ms for Alexa Fluor 488 used for all images. (A) Representative images are shown for the HepG2 cell line at 0 and 24 h for each of the treatment arms. Sample images for the other 3 cell lines are shown in Figure S3. The percent of nuclei demonstrating high (>25), moderate (10–25), low (<10), or no γ-H2AX foci was quantitated for each cell line at each time point by counting at least 5 representative high-power fields (HPF). The results of this quantitation are shown in graphical form with SEM for each treatment arm of each cell line (B–E). For all cell lines, radiation (RT) with or without SOR resulted in a significantly greater percent of nuclei with a high number of foci at t = 0 compared to the non-irradiated SOR and control arms. At t = 24 h, 3 of the 4 cell lines (HepG2, HCC-4-4, Hep3b) demonstrated a significantly greater percent of nuclei with a high number of persistent foci in the RT arm compared to the concurrent RT-SOR (CONC) arm (B–D). The fourth cell line (HuH7) showed no difference in number of nuclei manifesting any level of γ-H2AX foci between the RT and CONC treatment arms (E). Asterisks represent significant differences between treatment arms by Fisher's exact test as indicated by accompanying brackets. Dotted lines are merely included in cases of overlapping brackets to avoid confusion. Cover slips with treated/fixed cells were prepared in triplicate.

Based on the observed sorafenib-mediated effects on the cell cycle in three of the HCC cell lines we examined, we hypothesized that concurrent treatment with sorafenib may cause radioprotection by promoting reassortment into less radiosensitive phases of the cell cycle. To investigate this possibility, cells were incubated with sorafenib for 24 h, then irradiated with 6 Gy, fixed at time points of 30 minutes and 24 h, probed for γ-H2AX foci indicative of double-strand breaks (DSBs), and quantitated with a confocal microscope (Fig. 4A–E). Among all four HCC cell lines, irradiated cells exhibited a much greater proportion of nuclei with a high number (>25) of foci at 30 m following irradiation than cells treated with sorafenib or vehicle control alone, as expected (all p<0.001, Fisher's exact test). Treatment with radiation resulted in similar amounts of foci per nucleus at 30 m post-irradiation regardless of treatment with sorafenib, with almost all nuclei displaying a high number of foci after concurrent sorafenib-radiation or radiation alone (p>0.05 for all cell lines, Fisher's exact test). At 24 h post-irradiation, HuH7 cells did not exhibit significantly different proportions of nuclei with a high number of foci after concurrent sorafenib-radiation compared to radiation alone (p = 0.73). However, the remaining three HCC cell lines did demonstrate a greater proportion of nuclei with a high number of persistent foci after radiation alone versus concurrent sorafenib-radiation (p<0.001 for HepG2 and HCC-4-4; p = 0.02 for Hep3b, Fisher's exact test). Similar to the short term *in vitro* apoptosis assays conducted above we could not model sequential radiation-sorafenib effects on γ-H2AX foci given the rapid repair of DSBs. Despite this limitation, our data show that concurrent sorafenib-radiation treated HCC cells demonstrated reduced persistence of DSBs compared to HCC cells treated with radiation alone.

### Sequential treatment with radiation followed by sorafenib resulted in greater tumor growth delay *in vivo* than concurrent treatment with sorafenib and radiation

**Figure 5:**
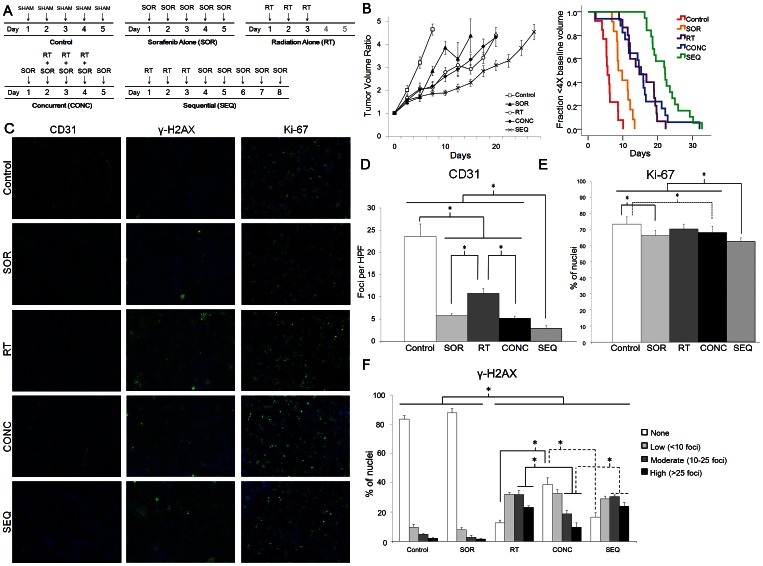
A sequential radiation-sorafenib regimen is most efficacious against HCC *in vivo*. A HepG2 hind-flank xenograft model was utilized to measure the efficacy of (A) 5 different treatment arms: control (sham injection of vehicle control on days 1–5), sorafenib alone (SOR; injection of 6 mg/mL sorafenib on days 1–5), radiation alone (RT; irradiation at a dose of 3 Gy on days 1–3), concurrent radiation-sorafenib (CONC; sorafenib injection on days 1–5 and irradiation at a dose of 3 Gy on days 2–4), and sequential radiation-sorafenib (SEQ; irradiation at a dose of 3 Gy on days 1–3 and sorafenib injection on days 4–8). The number of tumors per arm was: *n* = 13 for control, *n* = 12 for SOR, *n* = 15 for RT, *n* = 17 for CONC, and *n* = 19 for SEQ. Data for each arm are plotted as (B) tumor volume ratio over time (*left*) and as Kaplan-Meier curves with attainment of quadruple the pre-treatment tumor volume as the event of interest (*right*). Using two methods of statistical analysis (Mann-Whitney U-test for *left* and log-rank test for *right*), SEQ was shown to achieve a significantly longer time to quadruple the pre-treatment tumor volume than any of the other treatment arms. The CONC and RT arms were not significantly different from one another. (C–F) Immunofluorescence staining from xenografts harvested from all treatment arms show significantly more downregulation of vascularity (CD31) (C – *left column*, D) and decreased mitotic index (Ki-67) (C – *right column*, E) in arms that received sorafenib treatment, with the most pronounced reductions occurring in the SEQ arm. Immunohistochemical staining for γ-H2AX (C – *middle column*, F), however, revealed a significantly greater percent of nuclei with high or moderate numbers of foci, as well as a lower percent of nuclei with no foci, for the SEQ and RT arms compared to the CONC arm, similar to our *in vitro* results above. Column graphs summarizing the data for CD31, γ-H2AX, and Ki-67 are shown in D-F. Asterisks represent significant differences between columns ascertained by Student's *t-*test (CD31) or by Fisher's exact test (γ-H2AX and Ki-67) as indicated by the accompanying brackets.

To confirm that our *in vitro* observations were relevant to tumors in living organisms, HepG2 cells were implanted in the flanks of nude mice to examine the effects of concurrent and sequential treatment with radiation and sorafenib *in vivo* (Fig. 5A). At least 12 tumors were assessed for each treatment arm (Fig. 5B). The quadrupling time or time for tumors to reach 4× their starting size was 6.0 days (SD 2.1) for mice treated with vehicle control. All treatment arms demonstrated efficacy compared to control treatment with increased quadrupling times of 9.9 days (SD 2.2) for sorafenib alone, 15.3 days (SD 4.3) for radiation alone, 15.4 days (SD 6.6) for concurrent sorafenib-radiation, and 22.2 days (SD 4.7) for sequential radiation followed by sorafenib (Fig. S4; all p≤0.001, Mann-Whitney U-test). No significant difference in quadrupling time was observed between treatment with concurrent sorafenib-radiation and radiation alone (Fig. S4; p = 0.87, Mann-Whitney U-test). However, sequential treatment with radiation-sorafenib resulted in a quadrupling time that was significantly longer than that for concurrent sorafenib-radiation (Fig. S4; p = 0.0005, Mann-Whitney U-test) or radiation alone (Fig. S4; p = 0.001, Mann-Whitney U-test). To show the behavior of all tumors used in the analysis over time, the data are also represented using Kaplan-Meier statistics with quadrupling time as an event (Fig. 5B). All relationships noted above to be significant by the Mann-Whitney U-test were found to be similarly significant with Kaplan-Meier statistics compared by the log-rank test. HCC tumors treated with sequential radiation-sorafenib required an average of 16.2 (SD 4.7) days more to quadruple compared to untreated tumors (22.2 days−6 days = 16.2 days). This is similar to the sum of increases in HCC tumor growth delay seen with sorafenib alone and radiation alone (3.9 days+9.3 days = 13.2 days), which suggests that sequential treatment additively delays tumor growth. No weight loss, diarrhea, dermatitis, ulceration, or other observable toxicity was noted due to sorafenib, radiation, or either schedule of combined radiation-sorafenib treatment throughout these experiments.

### Sorafenib decreased tumor vascularity and mitotic index *in vivo* most markedly with sequential treatment

Immunofluorescence staining was performed on three tumors per treatment arm from our xenograft experiments. Tumor sections were stained for cluster of differentiation 31 (CD31; also known as platelet endothelial cell adhesion molecule 1 or PECAM-1) to detect tumor vasculature by identifying endothelial cells [34] (Fig. 5C,D), for antigen Ki-67 to identify cells undergoing proliferation [35] (Fig. 5C,E), and for γ-H2AX foci to identify degree of DNA damage. Time points for tumor harvesting were as follows: (a) control arm – after 3 consecutive daily sham injections; (b) sorafenib arm – after 3 consecutive daily injections of sorafenib; (c) radiation alone arm – after 2 consecutive daily fractions of 3 Gy; (d) concurrent arm – after 3 consecutive daily injections of sorafenib with concomitant 3-Gy fractions of radiation on days 2 and 3; (e) sequential arm – after 2 consecutive daily fractions of 3 Gy followed by 3 consecutive daily injections of sorafenib. In this way, animals in the concurrent and sequential arms received equal numbers of sorafenib and radiation doses.

Treatment with sorafenib alone markedly reduced the average number of blood vessels per high power field (HPF) from 23.6 (SD 6.2) for vehicle control to 5.8 (SD 0.8) (p = 0.0002, Student's t-test) (Fig. 5C,D). Treatment with radiation alone likewise reduced the average number of blood vessels per HPF, though to a lesser degree at 10.8 (SD 2.4). Concurrent sorafenib-radiation decreased the number of blood vessels per HPF to 5.2 (SD 1.2), a similar reduction to that observed with sorafenib alone (p = 0.54, Student's t-test). Therefore, concurrent sorafenib-radiation does not appear to significantly augment the reduction in vascularity beyond that achieved with sorafenib alone. However, sequential radiation-sorafenib significantly reduced tumor vascularity compared to all other arms at 2.9 (SD, 0.6) CD31 foci per HPF (all p<0.05). This evidence suggests that a sequential radiation-sorafenib schedule may additively enhance the anti-angiogenic effects of either radiation or sorafenib given alone, while a concurrent schedule may not.

Mitotic index as measured by Ki-67 was slightly lower for tumors treated with sorafenib alone (p = 0.002, Fisher's exact test) or concurrent sorafenib-radiation (p = 0.02 by Fisher's exact test) compared to untreated controls, whereas tumors treated with radiation alone showed no significant difference from untreated controls (p = 0.12, Fisher's exact test) (Fig. 5C,E). The mitotic index of tumors treated with sequential radiation- sorafenib was significantly lower than that for all other arms, including sorafenib alone and concurrent sorafenib-radiation- (all p<0.05).

### Concurrent treatment with sorafenib-radiation resulted in reduced persistence of double-strand breaks compared to treatment with sequential radiation-sorafenib or radiation alone *in vivo*

Immunofluorescence staining for γ-H2AX foci was assessed quantitatively and revealed that tumors treated concurrently with sorafenib-radiation displayed a lesser proportion of nuclei with high and moderate numbers of foci compared to tumors treated with sequential sorafenib-radiation or radiation alone (p<0.001 for both, Fisher's exact test) (Fig. 5C,F). Tumors treated with concurrent sorafenib-radiation also exhibited a greater proportion of nuclei with no foci compared to treatment with sequential sorafenib-radiation or radiation alone (p<0.001 for both, Fisher's exact test). These results are similar to our *in vitro* observations and suggest that sorafenib may have a cell-autonomous radioprotective effect on HCC cell lines *in vivo*. No significant differences in γ-H2AX foci distribution were observed between the radiation alone arm and the sequential arm.

## Discussion

Several ongoing phase I–II trials employ sorafenib and radiation concurrently, sequentially, or both (Table 1); however, preclinical data examining sorafenib and radiation in HCC that might guide selection of the optimal therapeutic sequence for clinical use is limited to one *in vitro* study, which did not involve any *in vivo* experiments [36]. To investigate the activity of combined sorafenib and radiation against HCC, we tested concurrent and sequential treatment approaches in four HCC cell lines. Using colony formation assays, we found that a sequential approach produced an additive increase in efficacy, while concurrent therapy resulted in radioprotection or no effect when compared to radiation alone, corroborating the results of Li and colleagues [36]. Based on previous work in non-HCC cell lines [29], we hypothesized that this effect of concurrent treatment might be due to sorafenib-mediated reassortment into less radiosensitive phases of the cell cycle and generalized cell cycle slowing. To test this hypothesis, mechanistic *in vitro* studies analyzing the degree of apoptosis, cell cycle progression, and repair of radiation-induced double-strand breaks (DSBs) were performed. These experiments showed that sorafenib caused cell cycle delay in diploid HCC cells and reduced persistent DSBs following radiation when given concurrently. We confirmed our *in vitro* results by testing concurrent and sequential regimens in a nude mouse HCC xenograft model. While *in vivo* studies showed concurrent treatment to be similar to radiation alone, sequential treatment using fractionated radiation followed by sorafenib clearly emerged as the ideal treatment approach. The preclinical data presented herein may serve to inform the interpretation of results from continuing phase I–II trials and the design of imminent phase III trials utilizing sorafenib and radiation against HCC.

Using a long-term clonogenic survival assay, our *in vitro* data demonstrated that concurrent sorafenib-radiation resulted in radioprotection of three of the four HCC cell lines examined. Sorafenib has likewise been shown to counteract the cytotoxic effects of other DNA damaging agents, such as platinum based chemotherapy [37]. Mechanistically, the cell-autonomous radioprotective effect we observed could be explained by sorafenib-mediated reassortment into cell cycle phases of relatively greater radioresistance and a generalized cell cycle slowing allowing increased time for sublethal damage repair. Classically, cells were observed to be most radioresistant during S phase and most radiosensitive during G2 and M phases, with intermediate radiosensitivity during G1 phase [38], [39]. More recent data suggest that radiosensitivity varies even within a given phase of the cell cycle, probably in part due to fluctuation in activity levels of different DNA damage repair pathways (*e.g.*, homologous recombination versus non-homologous end-joining) within individual phases [40]–[42]. What appears clear from these studies, however, is that cells are most radiosensitive during late G2 or M after they have passed the G2 checkpoint, likely as a result of reduced time to perform sublethal damage repair prior to mitotic catastrophe. Our data demonstrate that sorafenib consistently reduced the number of cells in G2 and M among the three diploid HCC cell lines examined. Sorafenib has been observed to exert a similar effect on the cell cycle in some non-HCC cell lines [29], which is not unexpected given its well-established inhibition of the pro-proliferative MAP kinase pathway [10], [11]. Therefore, a possible explanation for the radioprotection observed *in vitro* is that the proportion of cells in late G2 or M at the time of irradiation was reduced by treatment with sorafenib.

In a fashion similar to our *in vitro* findings, examination of treated HCC tumor xenografts showed that concurrent sorafenib-radiation therapy resulted in reduced persistence of double-strand breaks compared to radiation alone. We also observed a lower mitotic index among HCC cells in tumor xenografts following sorafenib treatment *in vivo*, recapitulating the reduced rate of proliferation seen *in vitro* by cell cycle analysis. These data suggest that the interaction between sorafenib and radiation in HCC cells may be similar *in vitro* and *in vivo*. In spite of these observations, no difference in efficacy was seen between concurrent sorafenib-radiation and radiation alone when tumor growth delay was measured *in vivo*. This lack of difference is unexplained by our study. However, one feasible hypothesis is that cell-autonomous radioprotective effects of sorafenib on tumor cells were balanced *in vivo* by additional anti-cancer non-cell-autonomous effects (such as antiangiogenic effects and normalization of blood flow) [43] that were unobservable *in vitro*, resulting in no net difference from radiation alone. Whatever the true mechanistic explanation may be, it remains interesting and instructive for present and future clinical trials that concurrent sorafenib-radiation *in vivo* was not superior to radiation alone in producing tumor growth delay, despite the fact that each treatment had clear efficacy when administered separately.

Using both clonogenic survival assays *in vitro* and the HCC tumor xenograft model *in vivo*, we observed sequential radiation-sorafenib to be a superior regimen compared to sorafenib alone, radiation alone and concurrent sorafenib-radiation. The results of our Annexin V/propidium iodide flow cytometric assay suggest that increased apoptosis may result from a sequential regimen compared to a concurrent regimen or either agent alone. More investigation, however, is required to elucidate the mechanistic underpinnings at play. In a living host, our immunofluorescence data suggests that possible mechanisms contributing to the longer tumor growth delay observed with a sequential regimen may include reductions in tumor vascularity and mitotic rate beyond those achievable with a concurrent regimen or either modality alone. Regardless of the specific mechanism of action, both our *in vitro* and *in vivo* data suggest that the most effective combination schedule for these agents is a sequential one. The ideal combined-modality approach to treating unresectable HCC may prove to involve radiotherapy administered concurrently with a radiosensitizing agent followed by maintenance sorafenib therapy. Although no targeted agents with well-established radiosensitizing activity against HCC have yet been identified, promising candidates include inhibitors of heat-shock proteins [44], EGFR [45], and the PI3K-AKT-mTOR pathway [46].

In summary, our study presents evidence that concurrent schedules of sorafenib-radiation may result in tumor control that is equal to or worse than with radiation alone. Moreover, sequential treatment with radiation followed by sorafenib appears to be more efficacious against HCC both *in vitro* and *in vivo* than either agent given alone or concurrently. These results have implications for clinical decision making as well as current and future trial design in HCC.

## Supporting Information

Figure S1 (A–C) Additional Annexin V-FITC and propidium iodide flow cytometry data. Representative data showing percent of cells in the early (quadrant II) or late (quadrant III) phases of apoptosis for the other 3 cell lines (HCC-4-4, Hep3b, and HuH7) after treatment with control, sorafenib (SOR), radiation (RT), concurrent therapy (CONC), or sequential therapy (SEQ) as delineated in the Methods and in the legend for Figure 2. All data in S1(A–C) were omitted from Figure 2 due to space constraints; please refer to the Figure 2 legend for details.
(TIF)

Figure S2 Additional cell cycle analysis data. Representative flow cytometry histograms are shown for the HuH7 cell line after treatment with sorafenib (SOR) and control, revealing several peaks on cell cycle analysis indicative of polyploidy (A). The effect of 24-h incubation with SOR versus control on unsynchronized HepG2 cells is shown as a column chart with SEM accompanied by representative flow cytometry histograms below (B). Unsynchronized HepG2 cells demonstrate a significantly greater proportion of cells in S phase and significantly fewer cells in G_1_ or G_2_-M after 24-h incubation with SOR. Asterisks indicate significant differences determined by Student's *t*-test. (C) Immunoblot data obtained upon probing the HCC-4-4, Hep3b, and HuH7 cell lines for phospho-p53 and p21 after treatment with one of the 5 treatment arms as delineated in the Methods and in the legend for Figure 2.
(TIF)

Figure S3 Additional γ-H2AX immunostaining fluorescent images. Fluorescent images for cell lines other than HepG2 were omitted from Figure 4 due to space constraints. The sample images from the HCC-4-4 (A), Hep3b (B), and HuH7 (C) cell lines are displayed here for each treatment arm at t = 0 and t = 24 h. All images were captured at 40× using a confocal microscope with uniform exposures of 50 ms for DAPI and 900 ms for Alexa Fluor 488. Please refer to the Figure 4 for graphical representation of the full dataset and for further details.
(TIF)

Figure S4 Additional column chart showing average number of days to quadrupling of pre-treatment tumor volume with SEM for each treatment arm. All treatment arms resulted in significantly longer time to quadrupling than untreated control tumors. Radiation alone (RT) and concurrent radiation-sorafenib (CONC) were not significantly different from one another, but were both superior to sorafenib alone (SOR). SEQ was significantly more effective than either RT or CONC. Asterisks and accompanying brackets represent significant differences by the Mann-Whitney U-test. This column chart was omitted from Figure 5 due to space constraints; please refer to the Figure 5 legend for further details.
(TIF)
