# Supplementary figures and images for: Concurrent versus Sequential Sorafenib Therapy in Combination with Radiation for Hepatocellular Carcinoma
Source: PLoS One. 2013 Jun 6;8(6):e65726. doi: 10.1371/journal.pone.0065726 (PMC3675179; doi:10.1371/journal.pone.0065726)

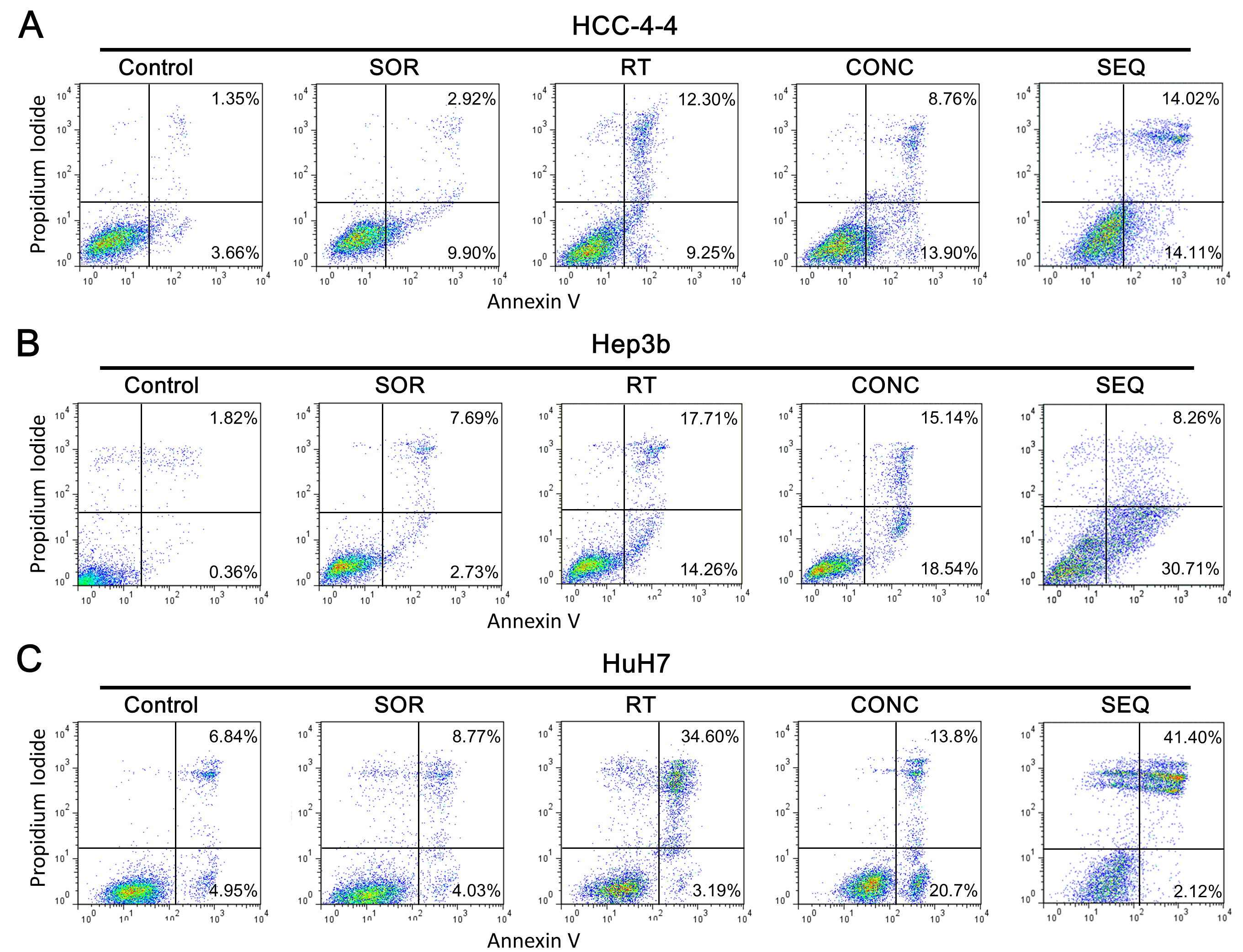

Supplement: Figure S1 — (A–C) Additional Annexin V-FITC and propidium iodide flow cytometry data. Representative data showing percent of cells in the early (quadrant II) or late (quadrant III) phases of apoptosis for the other 3 cell lines (HCC-4-4, Hep3b, and HuH7) after treatment with control, sorafenib (SOR), radiation (RT), concurrent therapy (CONC), or sequential therapy (SEQ) as delineated in the Methods and in the legend for Figure 2. All data in S1(A–C) were omitted from Figure 2 due to space constraints; please refer to the Figure 2 legend for details. (TIF) [file pone.0065726.s001.tif]

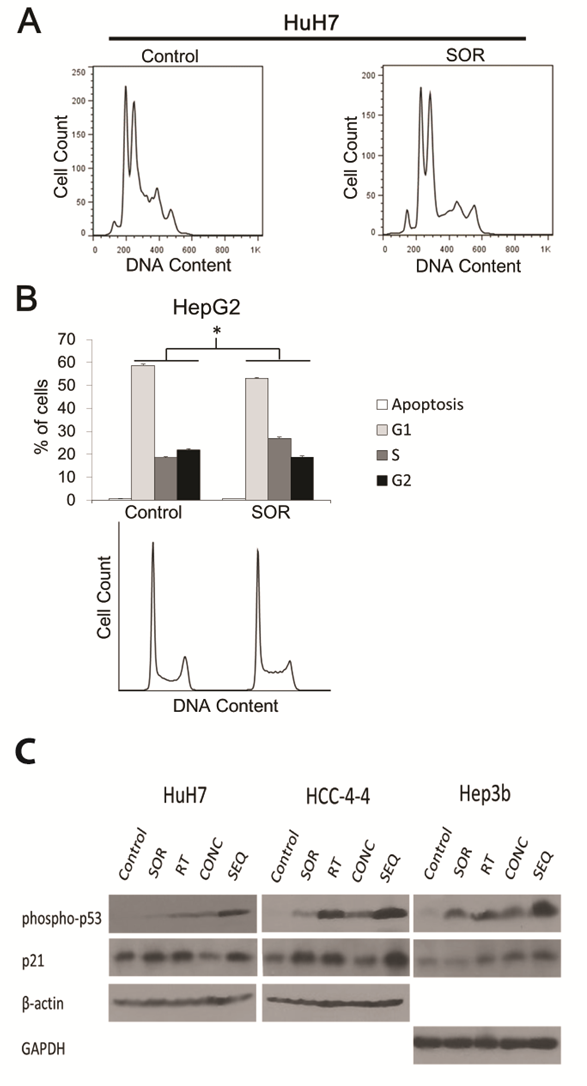

Supplement: Figure S2 — Additional cell cycle analysis data. Representative flow cytometry histograms are shown for the HuH7 cell line after treatment with sorafenib (SOR) and control, revealing several peaks on cell cycle analysis indicative of polyploidy (A). The effect of 24-h incubation with SOR versus control on unsynchronized HepG2 cells is shown as a column chart with SEM accompanied by representative flow cytometry histograms below (B). Unsynchronized HepG2 cells demonstrate a significantly greater proportion of cells in S phase and significantly fewer cells in G1 or G2-M after 24-h incubation with SOR. Asterisks indicate significant differences determined by Student's t-test. (C) Immunoblot data obtained upon probing the HCC-4-4, Hep3b, and HuH7 cell lines for phospho-p53 and p21 after treatment with one of the 5 treatment arms as delineated in the Methods and in the legend for Figure 2. (TIF) [file pone.0065726.s002.tif]

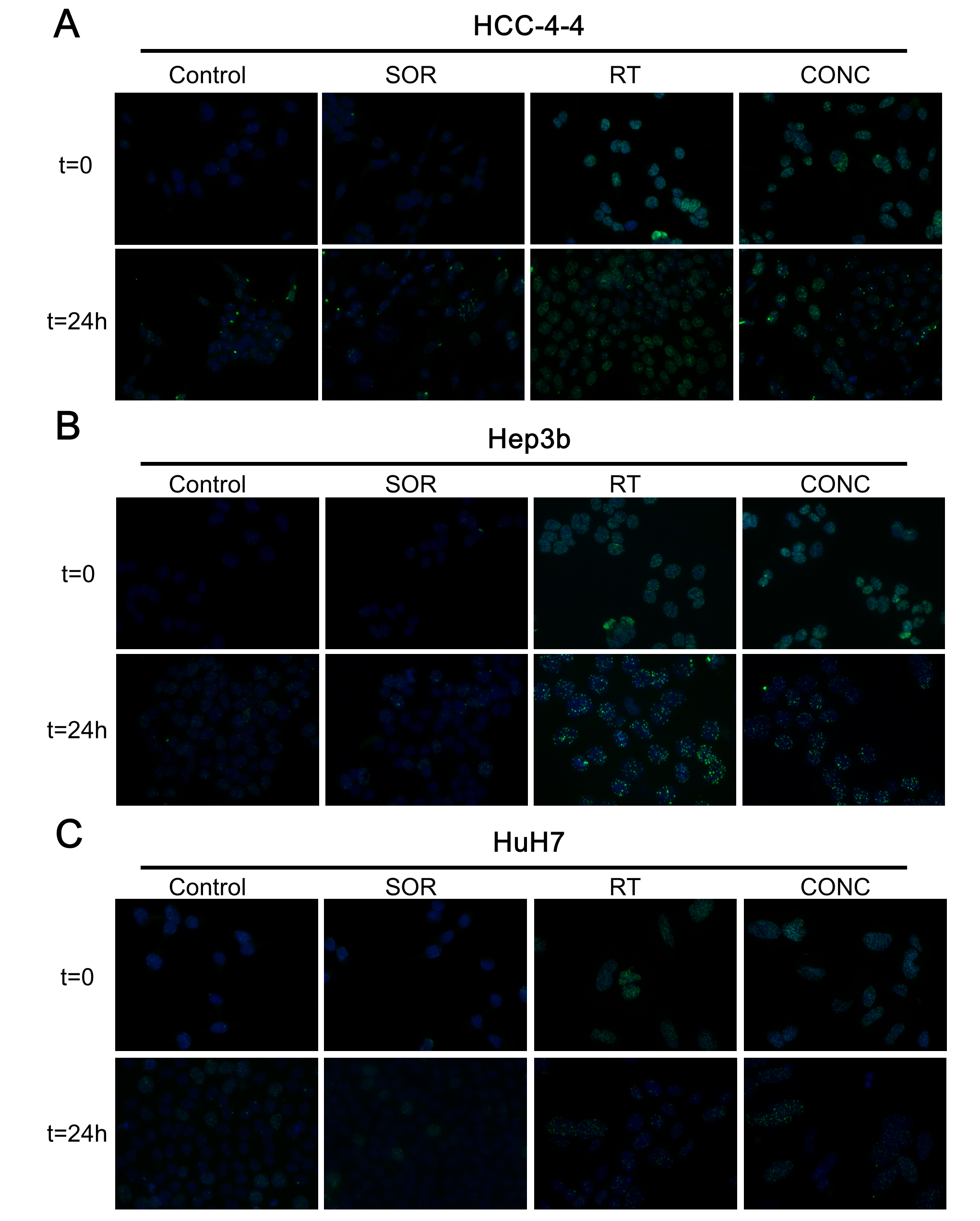

Supplement: Figure S3 — Additional γ-H2AX immunostaining fluorescent images. Fluorescent images for cell lines other than HepG2 were omitted from Figure 4 due to space constraints. The sample images from the HCC-4-4 (A), Hep3b (B), and HuH7 (C) cell lines are displayed here for each treatment arm at t = 0 and t = 24 h. All images were captured at 40× using a confocal microscope with uniform exposures of 50 ms for DAPI and 900 ms for Alexa Fluor 488. Please refer to the Figure 4 for graphical representation of the full dataset and for further details. (TIF) [file pone.0065726.s003.tif]
